# Calibration of Minimally Invasive Continuous Glucose Monitoring Sensors: State-of-The-Art and Current Perspectives

**DOI:** 10.3390/bios8010024

**Published:** 2018-03-13

**Authors:** Giada Acciaroli, Martina Vettoretti, Andrea Facchinetti, Giovanni Sparacino

**Affiliations:** Department of Information Engineering, University of Padova, 35131 Padova, Italy; giada.acciaroli@phd.unipd.it (G.A.); vettore1@dei.unipd.it (M.V.); facchine@dei.unipd.it (A.F.)

**Keywords:** diabetes, glucose sensors, continuous glucose monitoring, calibration

## Abstract

Minimally invasive continuous glucose monitoring (CGM) sensors are wearable medical devices that provide real-time measurement of subcutaneous glucose concentration. This can be of great help in the daily management of diabetes. Most of the commercially available CGM devices have a wire-based sensor, usually placed in the subcutaneous tissue, which measures a “raw” current signal via a glucose-oxidase electrochemical reaction. This electrical signal needs to be translated in real-time to glucose concentration through a calibration process. For such a scope, the first commercialized CGM sensors implemented simple linear regression techniques to fit reference glucose concentration measurements periodically collected by fingerprick. On the one hand, these simple linear techniques required several calibrations per day, with the consequent patient’s discomfort. On the other, only a limited accuracy was achieved. This stimulated researchers to propose, over the last decade, more sophisticated algorithms to calibrate CGM sensors, resorting to suitable signal processing, modelling, and machine-learning techniques. This review paper will first contextualize and describe the calibration problem and its implementation in the first generation of CGM sensors, and then present the most recently-proposed calibration algorithms, with a perspective on how these new techniques can influence future CGM products in terms of accuracy improvement and calibration reduction.

## 1. Introduction

Diabetes is a chronic disorder that occurs either when the pancreas is no longer able to produce insulin (type 1 diabetes, T1D), or if body tissues and organs cannot effectively utilize circulating insulin (type 2 diabetes, T2D). In people with diabetes, the deficiencies in glucose control lead to blood glucose (BG) values exceeding the safe range of 70–180 mg/dL. While hyperglycemia (i.e., BG > 180 mg/dL) can result in long-term complications, e.g., retinopathy, nephropathy, cardiovascular disease, hypoglycemia (i.e., BG < 70 mg/dL) can produce short-term adverse conditions that can cause coma, or even death [1,2,3,4].

Nowadays, diabetes afflicts more than 350 million people worldwide. T2D accounts for about 90% of all cases [5] and its onset is largely correlated with excessive body weight, physical inactivity, and unhealthy diet. The World Health Organization (WHO) predicts T2D prevalence to significantly grow in the coming years, due to aging populations and sedentary lifestyles [6,7]. Consequently, the total number of cases of diabetes is expected to exceed 500 million by 2030, becoming one of the most challenging socio-health emergencies of the third millennium. Although it is not possible with current knowledge to definitely cure diabetes, a constant and appropriate management of the disease can control and prevent many complications [6,8,9]. While T2D management mainly consists of healthy diet, physical exercise, and drug administration, T1D therapy requires daily insulin administration. The most challenging issue is related to insulin administration, being the correct amount of injected insulin determined on the basis of BG concentration levels, which need to be monitored by suitable technologies.

**Figure 1:**
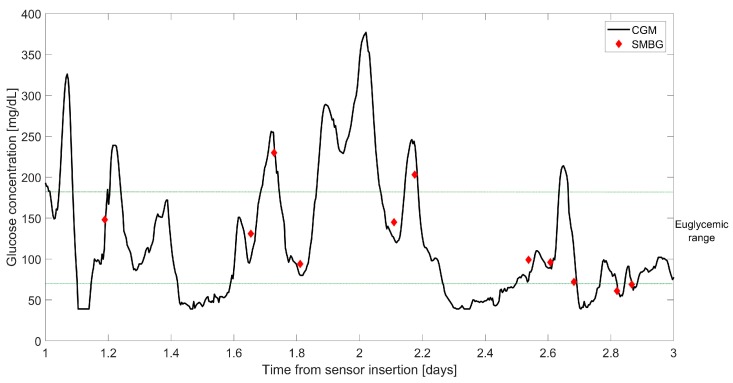
Representative three days of blood glucose (BG) monitoring obtained with self-monitoring BG (SMBG), diamonds, and with continuous glucose monitoring (CGM), continuous line. Horizontal dashed lines indicate the euglycemic range. Data taken from a previously published study [15].

At-home BG monitoring became available only in the 1970s, when the first self-monitoring BG (SMBG) meters were commercialized [10]. The early SMBG portable devices were based on optical detection of a color change on glucose oxidase-based strips, while the most recent systems rely on electrochemical-based sensing techniques [11] becoming, by the mid-1980s, the landmark in diabetes management [10]. Since the advent of SMBG devices for home BG testing, the standard therapy for diabetes management consisted of 3–4 SMBG measurements per day, individually acquired by fingerprick tests [6]. Although the introduction of self-BG monitoring in everyday life resulted in a general improvement of metabolic control [12,13,14], sparse BG measures cannot provide a complete description of glucose dynamics during the day. For instance, hypoglycemic or hyperglycemic events occurring between two BG acquisitions cannot be detected. An example of SMBG time series (data derived from a previously published study [15]) is depicted in Figure 1 (diamonds), where it is apparent that some critical episodes, e.g., a hypoglycemia event and several hyperglycemic conditions, cannot be revealed due to insufficient sampling frequency. On the one hand, more frequent SMBG measurements would be required to optimize glucose control but, on the other hand, this would increase the patient’s discomfort and increase the number of actions needed daily to manage the disease.

In recent years, technological innovations have been introduced for the treatment of diabetes. In particular, the development of continuous glucose monitoring (CGM) sensors [16,17] have revolutionized diabetes management. CGM systems are wearable devices able to measure subcutaneous glucose concentration almost continuously, e.g., every 1–5 min [18], for several consecutive days, greatly increasing the information on glucose dynamics compared to standard SMBG-based monitoring, with consequent improvement of glycemic control, quality of life, and reduction of diabetes-related complications [19,20,21,22,23,24,25,26]. See, for instance, in Figure 1 (continuous line) the hypo- and hyperglycemic episodes detected by the Dexcom G4 Platinum (Dexcom Inc., San Diego, CA, USA) CGM sensor (data previously published in [15]).

A variety of sensing techniques (e.g., electrochemical, optical, piezoelectric) have been proposed for CGM [27,28,29,30,31,32,33,34,35], but most of the devices currently available on the market exploit the glucose-oxidase electrochemical principle [18,36]. In this family of devices, a minimally-invasive wire-based sensor, placed subcutaneously in the abdomen or in the arm, measures a current signal generated by the glucose-oxidase reaction, transmitting information on glucose concentration in the interstitial fluid. The raw current sensor signal (typically measured in fractions of ampere) is converted to a glucose concentration estimate (expressed in mg/dL) by a calibration process. Usually, the calibration algorithm relies on a calibration function, whose parameters are periodically updated using a few SMBG samples suitably collected by the patient as reference measurements [37,38,39,40,41,42].

Most commercialized minimally-invasive CGM systems perform the first calibration a few hours (e.g., one or two) after sensor insertion, when the sensor warm-up period has completed, and the subsequent ones every 12–24 h, usually employing a simple first-order time-independent linear function as the calibration law [37,38,39,40]. Given the complex nonlinear and time-dependent relationship between measured current and glucose concentration, the use of a simple linear function, as an approximation of the more complex behavior, is acceptable within time-intervals of limited duration. Thus, frequent recalibrations are required to maintain sensor accuracy, as recommended by the manufacturers’ instructions [41,42,43]. Patients’ discomfort associated to the frequent calibration of the device on the basis of SMBG fingerprick measurements, and the need to improve CGM sensors’ accuracy and reliability called for the development of more sophisticated calibration techniques. In the last decade, several signal processing, modelling, and machine-learning methods have been proposed by the academic community to address the calibration issue, which led to improvements in CGM sensor accuracy and user acceptability. The aim of the present paper is to review the calibration algorithms proposed for minimally-invasive CGM sensors with a perspective on how these new techniques can influence future CGM products.

## 2. Calibration of Minimally-Invasive CGM Sensors

### 2.1. Problem Statement

CGM sensors measure a signal that reflects glucose concentration only indirectly. Indeed, the needle placed in the subcutaneous tissue measures a current signal derived from the glucose-oxidase electrochemical reaction [11,36]. The calibration process consists in the estimation of a mathematical law that converts the current signal (given in fractions of ampere) into meaningful glucose concentration values (in mg/dL).

Letting x(t) be the glucose concentration profile, y(t) the electrical current profile, and f(·) the function of parameters $P=\left[p_{1},p_{2},\ldots ,p_{n}\right]$ that relates x(t) and y(t), the calibration process can be schematized in two steps. The first consists in the identification of the calibration parameters. In formal terms, the current signal y(t) collected by the sensor and corrupted by measurement error e(t), and the BG measurements (samples of x(t)) acquired by the patient at correspondent time instants, are described by the model:

(1) y(t)=f(P,x(t))+e(t)

from which a numerical value $\hat{P}$ of the calibration parameter vector can be provided using, for instance, parametric estimation techniques. This step can be repeated each time a new BG reference is available, with consequent updates of the calibration parameters $\hat{P}$ (e.g., every 12–24 h by acquiring SMBG samples). The second step leads to the estimation of the glucose concentration profile. Formally, from the vector of estimated parameters $\hat{P}$ and the measured current profile y(t), the calibrated glycemic profile $\hat{x}\left(t\right)$ is obtained in real-time by inverting the function f(·):

(2) $$\hat{x}\left(t\right)=f^{-1}\left(\hat{P},y\left(t\right)\right)$$

The choice of the calibration law f(·) is critical. It has to be invertible and it has to precisely describe the relation between the electrical current signal and glucose concentration, which can be, in the most general case, non-linear and time-variant (in this case, time *t* would be, explicitly, an input of *f*(·). Moreover, the choice of using either the electrical current or the BG measurements as independent variables in the calibration law may affect the calibration performance [44].

The most common and simplest calibration law adopted by manufacturers of CGM systems is a first-order time-independent linear function [45,46,47,48], with parameters P=[s,b], where s and b are referred to as sensor sensitivity and baseline (or offset), respectively. In this case, the model of the measurements reported in Equation (1) in a general form turns into:

(3) y(t)=f(P,x(t))=s·x(t)+b+e(t)

The numerical determination of the estimates $\hat{s}$ and $\hat{b}$ is, thus, required. For such a scope, if for instance two BG references $x\left(t_{1}\right)$ and $x\left(t_{2}\right)$ are available at times $t_{1}$ and $t_{2}$, knowing the electrical current values given by the sensor at the same time instants, $y\left(t_{1}\right)$ and $y\left(t_{2}\right)$, the so-called two-point calibration can be performed [49], which allows the estimation of sensitivity, $\hat{s}$, and baseline, $\hat{b}$, from the two measured pairs as:

(4) $$\{\begin{array}{c} \hat{s}=\frac{y\left(t_{2}\right)-y\left(t_{1}\right)}{x\left(t_{2}\right)-x\left(t_{1}\right)}\\ \hat{b}=y\left(t_{2}\right)-\frac{y\left(t_{2}\right)-y\left(t_{1}\right)}{x\left(t_{2}\right)-x\left(t_{1}\right)}\cdot x\left(t_{2}\right) \end{array}$$

In general, when multiple pairs of electrical current and BG samples are available at times $t_{i}$ (i=1, 2…, N), a linear regression is used to fit the sensitivity and baseline to the data. In particular, including the measurement noise $e\left(t_{i}\right)$, the model of the measurements becomes:

(5) $$y\left(t_{i}\right)=s\cdot x\left(t_{i}\right)+b+e\left(t_{i}\right)$$

and the numerical determination of model parameters is done by minimizing the residual sum of squares:

(6) $$\left[\hat{s},\hat{b}\right]=\underset{s,b}{\mathrm{argmin}}\overset{N}{\underset{i=1}{\sum }}e{\left(t_{i}\right)}^{2}$$

Finally, the calibrated glucose profile $\hat{x}\left(t\right)$ is obtained from the measured current signal y(t) and the estimated calibration parameters $\hat{s}$ and $\hat{b}$ by inverting the calibration function:

(7) $$\hat{x}\left(t\right)=\frac{y\left(t\right)-\hat{b}}{\hat{s}}$$

The quality of the estimate of the calibration parameters is expected to increase with N, i.e., the more electrical current-BG pairs that are available, the more accurately the calibration parameters are estimated. On the other hand, increasing N is difficult to satisfy, for practical reasons, e.g., for the discomfort related to the acquisition of SMBG samples, and because CGM manufacturers push to minimize the calibration points to facilitate the ease-of-use of their devices. Moreover, in the presence of measurement uncertainty and/or when only a few data points are available, the standard two-point calibration of Equation (4) could be simplified to a one-point calibration by considering a zero baseline. This simplification may improve the calibration performance by reducing the effect of the noise [49,50].

**Figure 2:**
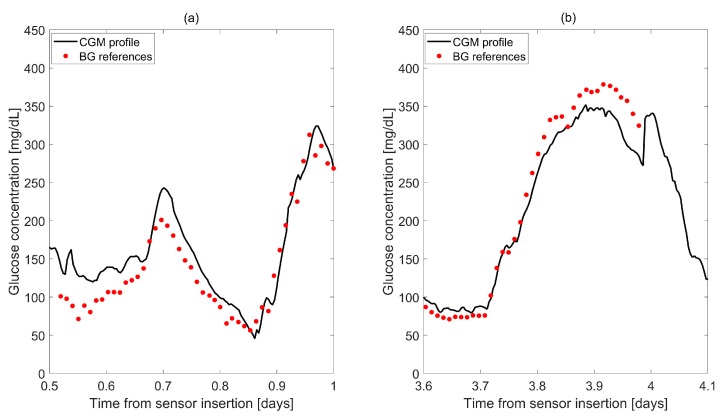
Examples in which the continuous glucose monitoring (CGM) sensor output (continuous line) (**a**) overestimates and (**b**) underestimates the reference blood glucose (BG) (points). Data taken from a previously published study [15].

Although the use of such linear calibration techniques is appealing for its simplicity and ease of implementation, it introduces several critical aspects that, together with uncertainty on the measured sensor output and BG references, are often a cause of CGM sensor inaccuracy. Two examples of sensor inaccuracy are illustrated in Figure 2a,b, where two representative CGM profiles (continuous lines) acquired by the Dexcom G4 Platinum (Dexcom Inc., San Diego, CA, USA) CGM sensor (data previously published in [15]) are compared with reference BG concentrations measured in parallel by gold-standard laboratory instruments (points). Two major causes of these deviations are discussed in the following section.

### 2.2. Critical Aspects Affecting Calibration 

**Figure 3:**
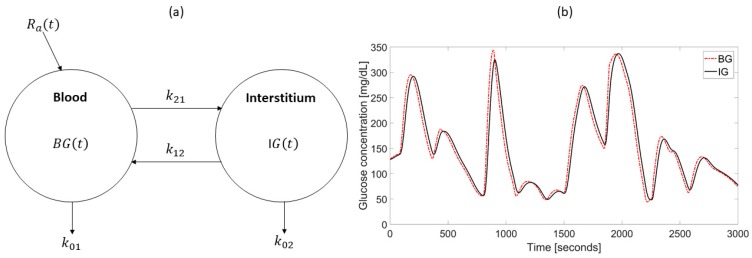
(**a**) Two-compartment model describing the blood glucose to interstitial glucose (BG-to-IG) kinetics. $R_{a}$ is the rate of appearance; $k_{01},k_{02},k_{12},k_{21}$ are rate constants. The time constant of the BG-to-IG system is $\tau =\frac{1}{k_{02}+k_{12}}$. (**b**) Representative blood glucose (BG) (dashed line) and interstitial glucose (IG) (continuous line) concentration profiles simulated as described in the text assuming τ = 11 min.

A first aspect explaining the discrepancies evidenced in Figure 2 is the distortion introduced by the plasma to interstitium (BG-to-IG) kinetics. Indeed, the needle sensor is inserted in the subcutis and measures a current that is proportional to the interstitial glucose (IG). This is due to the fact that, in order to reduce invasiveness of CGM devices, sensors are placed in the subcutis and measure the glucose-related current signal from the interstitial fluid rather than directly from the blood. Thus, the two measurements available during the calibration process, i.e., the electrical current signal measured by the sensor and the BG references acquired through fingerprick devices, belong to different physiological sites. A widely-established description of the relationship between BG and IG is based on a two-compartment model (Figure 3a) [51,52]. According to this representation, and noting that, in steady-state, BG and IG have equal values, the IG profile can be described by the convolution of the BG profile with a single exponential $\frac{1}{\tau }e^{-\frac{t}{\tau }}$, a decay function having unitary area under the curve and time constant τ The time constant τ is related to the parameters of the two-compartment model by the equation $\tau =\frac{1}{k_{02}+k_{12}}$ [51,52]. Given the low-pass filtering nature of the system described in Figure 3a, the IG signal is a smoothed and delayed version of the BG concentration [53]. An example is reported in Figure 3b, where the IG profile (obtained by convoluting a given, simulated, BG profile with a single exponential with τ = 11 min) clearly shows both amplitude attenuation and phase delay compared to the BG profile. Notably, τ shows inter- and intra-subject variability and its numerical identification requires suitable collection of both BG and IG samples. Published values of the time constant τ range from 6 to 15 min [52]. In practice, the BG-to-IG time constant τ is treated as a user parameter, but its role in the calibration process needs to be carefully considered [54,55].

**Figure 4:**
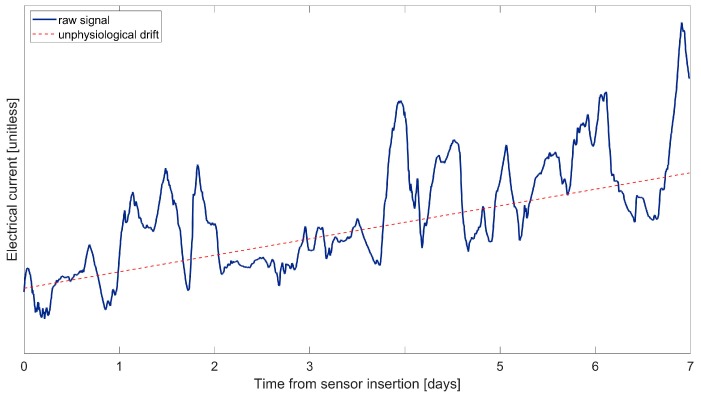
Representative raw CGM sensor signal (continuous line, units not specified by the manufacturer) that exhibits a nonphysiological drift (dashed line) due to the time-variability of sensor sensitivity. Data were previously published in [15].

A second critical aspect behind the differences pointed out in Figure 2 is related to the time variability of sensor sensitivity. The raw electrical current signals acquired by CGM sensors often exhibit a nonphysiological drift, especially in the first day after sensor insertion. An example of nonphysiological drift observed in a raw CGM signal acquired by the Dexcom G4 Platinum (Dexcom Inc., San Diego, CA, USA) CGM sensor (data previously published in [15]) is depicted in Figure 4, where the continuous line represents the electrical current signal (in units not specified by the manufacturer) and the dashed line shows the drift. This phenomenon is related to a variation of sensor sensitivity after its insertion in the body, when the sensor membrane enters in contact with the biological environment and undergoes the immune system reaction [56,57]. The calibration law has to properly compensate for such time-variability, which is often non-linear.

In the last decade, several techniques have been proposed to deal with these issues affecting CGM sensor calibration. A review and discussion of the most recent algorithms is presented in the following section.

## 3. State-of-the-Art Calibration Algorithms

One of the major limitations of the calibration linear regression techniques presented in Section 2 is that they all neglect the time lag between the BG and the raw sensor signal, which can lead to a suboptimal estimation of the parameters of the calibration function. Therefore, most of the calibration algorithms developed by the scientific community included more or less sophisticated approaches to overcome this limitation and take BG-to-IG dynamics into account.

The first simple approach is to require calibration of the sensor when glucose is relatively stable. This approach can be applied to any calibration algorithm. The rationale of this heuristic is that, in such a condition, BG and IG concentrations should be at equilibrium and, thus, the estimation of the linear regression parameters should not be influenced by not considering the BG-to-IG dynamics [58]. Following this rationale, Aussedat et al. [59] developed an automated algorithm that requests sensor calibration only when a window of the stable signal is detected, i.e., when the sensor signal has not changed by more than 1% over a four-minute window, and when the raw current value for the second calibration point differs from the first by ≥2 nA. The study proved that performing calibrations during periods of relative glucose stability minimizes difference between BG references and raw sensor measurements due to the BG-to-IG kinetics.

More sophisticated model-based approaches to account for the BG-to-IG dynamics have been developed relying on Kalman filter theory. In particular, Knobbe et al. [60] proposed a five-state extended Kalman filter, which estimates subcutaneous glucose levels, BG levels, time lag between the sensor measured subcutaneous glucose and BG, time-rate-of-change of the BG level, and the subcutaneous glucose sensor scale factor [61]. In this study, BG levels are reconstructed in continuous time from CGM measurements, employing a state-space Bayesian framework with a priori knowledge of unknown variables. A direct application of a Kalman filter to improve CGM sensor accuracy was proposed by Kuure-Kinsey and colleagues [62], employing a dual-rate Kalman filter and exploiting sparse SMBG measurements to estimate the sensor sensitivity in real-time. Although designed for real-time glucose and its rate of change estimation, the algorithm does not account for BG-to-IG kinetics. A further development, with direct application to the calibration problem and incorporation of the BG-to-IG dynamic model, was given by Facchinetti et al. [63]. The authors proposed an extended Kalman filter method that works in cascade to the standard device calibration to enhance sensor accuracy. By taking into account BG-to-IG kinetics, using a model to describe the variability of sensor sensitivity, and exploiting four BG reference samples per day, the method significantly improves CGM accuracy when applied to synthetic data. However, its real-time implementation is not straightforward, requiring the knowledge of the variances of both state and measurement error processes, as well as an initial burn-in interval.

Another approach for real-time glucose estimation based on autoregressive (AR) models was proposed by Leal et al. [64]. The study used AR models to estimate BG from raw CGM measurements. Data acquired from 18 T1D patients were used to train a population AR model, which was then incorporated into a calibration algorithm for real-time BG estimation. The raw sensor signal, used as the independent variable, and the BG concentration, considered as a dependent variable, were both normalized based on the maximum range of the available signals. The best overall estimated model, with a third-order Box-Jenkins structure and fixed parameters, enhanced CGM performance, especially in hypoglycemia detection. Significant improvement in hypoglycemia detection was also obtained by the same authors in another study performed on 21 patients with T1D where a new linear regression algorithm with enhanced offset estimation was proposed [65].

Barceló-Rico and colleagues proposed an alternative calibration algorithm based on a dynamic global model of the relationship between BG and the interstitial CGM signal [66]. The algorithm integrates several local dynamic models, each one representing a different metabolic condition and/or sensor–subject interaction. The local models are then weighted and added to compose the global calibration model. Inputs of the model are the signal measured by the sensor and other signals containing information relevant to glucose dynamics, which are normalized in magnitude using population parameters. The algorithm showed improvements in CGM sensor accuracy, although it was tested on only eight healthy subjects and a more extensive assessment on the diabetic population would be needed to confirm the findings. A further development of the algorithm was proposed in [67], where an adaptive scheme is used to estimate patient’s normalization parameters in real-time instead of using simple population parameters. Results on 30 virtual patients showed that the adaptation of normalization parameters further improved the performance of the algorithm, since they were able to compensate for sensor sensitivity variations.

Most of the algorithms proposed for improving CGM performance employ sophisticated models and signal processing features that, although still allowing the implementation on wearable devices/smartphones, increase the computational complexity and processing delay compared to the simple linear regression techniques. With the aim of reducing the delay due to signal processing, Mahmoudi et al. proposed a multistep calibration algorithm based on rate-limiting filtering, selective smoothing, and robust regression [68]. The rate-limiting filter limits the rate-of-change if a physiological threshold is exceeded; the selective smoothing is applied if the signal is noisy, i.e., if the number of zero crossings of the signal first-order differences exceeds a predefined threshold; the robust regression then converts the raw measured current to BG levels using reference SMBG measurements (for a maximum of four references per day). The application of the filtering step to only the noisy parts of the signal lowered the delay introduced by the signal processing of the CGM profile.

Another approach that has the low computational complexity as a major strength was proposed by Kirchsteiger and colleagues employing linear matrix inequalities techniques, resulting in convex optimization problems of low complexity [69,70]. The authors proposed two different parametric descriptions of the relationship between IG and BG and a constructive algorithm to adaptively estimate the unknown parameters. The algorithm explicitly considers the measurement uncertainty of the device used to collect the calibration measurements, which was firstly pointed out by Choleau and colleagues [71]. Moreover, the algorithm embeds an automatic feature to detect fingerprick measurements, which are not suitable to be used for calibration.

The uncertainty in the reference SMBG samples used for calibration is a key issue in the development of robust calibration algorithms. The real-time deconvolution-based approach proposed by Guerra et al. [72] demonstrated its robustness against both temporal misplacement of the SMBG references and uncertainty in the BG-to-IG kinetics model. The authors proposed a real-time signal-enhancement module to be applied to the CGM sensor output to improve the accuracy of the device. The algorithm compensates the distortion due to the BG-to-IG dynamic by means of regularized deconvolution [73] and relies on a linear regression model that is updated each time a pair of SMBG references is collected. Significant accuracy improvements were observed both on simulated and real datasets. The deconvolution-based approach of [72] was further developed in [74], where it was directly applied to the raw measured signal rather than in cascade to the CGM sensor output. The algorithm fits the raw current signal against BG references (collected twice a day) using a time-varying linear calibration function whose parameters are identified in the Bayesian framework using a priori knowledge on their statistical distribution. The BG-to-IG kinetics is compensated, as in [72], via nonparametric deconvolution. Results showed significant accuracy improvements compared to the manufacturer calibration.

The calibration algorithms discussed so far showed several performance improvements compared to the simple linear regression methods described in Section 2. However, none of them explicitly aimed at reducing the frequency of calibrations, i.e., the number of SMBG fingerprick measurements needed as input to the algorithms, which are an obvious reason of discomfort for the patients. At the present time, CGM systems require a calibration about two times per day, as per the manufacturer’s instructions. A first step toward the reduction of calibration frequency was made in [75]. The authors applied the same calibration strategy, as in [74], but employing day-specific prior calibration parameters that allowed the improvement of sensor performance (mean absolute relative difference, MARD, reduced by 1.2%), especially in the most critical first day of use, while, at the same time, reducing the frequency of calibrations, from twice, to once per day. The study demonstrated that, by formulating the calibration problem in the Bayesian framework, the use of well-tuned priors on calibration parameters can surrogate the information of a second daily SMBG reference while preserving sensor accuracy.

Although showing promising results in terms of accuracy enhancement and calibration frequency reduction, the linear calibration models used in [74,75] are able to approximate the time-variability of the relationship between the raw current signal and IG only for a time interval of limited duration. Thus, it is not suitable for further reducing the calibrations to less than once per day. To overcome this limitation, Acciaroli et al. [76] replaced the time-invariant sensitivity and baseline conventionally used by linear calibration models (see Equation (3)) with more sophisticated time-varying functions, valid for multiple-day periods, with unknown parameters for which an a priori statistical description is available. Calibration parameters are determined online by means of Bayesian estimation and BG-to-IG kinetics are compensated by nonparametric deconvolution. The method showed improved performance compared to manufacturer calibration (MARD reduced by 1.2%) with only two calibrations over the seven days of the sensor’s lifetime instead of twice per day.

Current CGM products are available for continuous use and are replaced after several days. However, none of the methods discussed so far have embedded any features able to capture this essential cyclic nature by exploiting, e.g., the data from prior weeks to better calibrate new CGM data. A first attempt in this direction was made by Lee and colleagues in [77], where a run-to-run strategy that personalizes sensor calibration parameters using data from previous weeks’ use was proposed. Before each weekly new sensor insertion, the algorithm minimizes a cost function that penalizes differences between fingerprick reference values and CGM output of previous weeks. Repeated iterations of the run-to-run procedure demonstrated improved performance on synthetic data (summed square error reduced by 20% after two weeks, and up to 50% after six weeks). On the same line, another calibration algorithm, employing a calibration function as in [74], was augmented with a weekly updating feature for parameter optimization [78]. The algorithm estimates the calibration parameters through the recursive least squares to fit SMBG measurements taken approximately every 12 h. Then, personalized calibration parameters are optimized after the first week of use using past data, employing a forgetting factor to give more weight to the most recent data.

The idea of exploiting past CGM data to optimize calibration has also been exploited for developing offline techniques able to improve the quality of the BG estimations through “retrospective” calibration. For instance, Hovorka et al. [79] proposed two algorithms for CGM-based trial assessment. The first is an offline retrospective CGM adjustment, and the second attempts to reduce CGM error by accounting for the possibility that the true BG could be in a different range compared to the CGM output. Del Favero and colleagues [80] proposed a “retrofitting” algorithm based on constrained deconvolution to retrospectively increase the accuracy of CGM data acquired on diabetic patients by using some BG reference measurements. The method proved effective when applied to different commercial products [81,82].

A summary of all revised techniques is reported in Table 1, in which, from column one to column seven, we reported the study reference; the calibration technique; whether a model of the BG-IG kinetic is used; whether the algorithm is suitable for real-time use in wearable devices; the number of calibrations required; the data on which the algorithms were validated; and the improvements compared to manufacturer performance (if available). It is worth noting that most of the proposed algorithms, although employing sophisticated signal processing techniques, are suitable for real-time use in the processors of CGM receivers or smartphones, or in dedicated cloud platforms. Moreover, where available, the comparison with the manufacturer calibration algorithm always showed enhanced performance and/or a reduction in the frequency of calibrations, improving sensor ease-of-use and reliability.

## 4. Current Perspectives 

In the past decade, the employment of state-of-the-art signal processing, modelling, and machine learning techniques proved effective in enhancing CGM sensor performance. Moreover, several proposed algorithms gave particular attention to the computational complexity, facilitating their implementation and integration in CGM wearable devices.

The first example of direct implementation of a newly-proposed technique in a commercial CGM product is described in [84], where Dexcom Inc. (San Diego, CA, USA) and the University of Padova (Padova, Italy) have developed an advanced CGM system, called the G4AP, containing updated denoising and calibration algorithms for improved sensor accuracy and reliability. It is likely that more algorithms will be included in next-generation CGM products, not only to improve the calibration performance, but also to integrate more sophisticated “smart” features, e.g., prediction and alert generation features [85].

Along with the need to constantly improve sensor performance, the need to reduce the frequency of calibration is among the priorities of CGM sensor manufacturers, as witnessed by the recent commercialization of the FreeStyle Libre (Abbott Diabetes Care, Alameda, CA, USA), a factory-calibrated flash glucose monitor [86]. Next-generation CGM systems are, thus, expected to be less calibration dependent, and the implementation of sophisticated modeling and signal processing techniques would eventually play a role in accomplishing such a requirement. A recent study conducted on a next-generation Dexcom CGM sensor prototype [83] showed that the implementation of the Bayesian calibration approach of [76] allowed moving toward a calibration-free scenario.

Factory-calibrated sensors appear to be the next future technology for both commercial and practical reasons, i.e., patients are not required to buy and deal with both the CGM and the fingerprick devices. Additionally, CGM technology has been recently approved for nonadjunctive use [87], i.e., treatment decisions can be made without fingerpricking. However, SMBG samples are still required by current commercial CGM systems for calibration purposes. Thus, on the one hand, the use of factory-calibrated CGM sensors as nonadjunctive devices would allow to definitively give up fingerpricking. On the other hand, factory-calibrated devices would probably lead to higher sensor bias and this aspect needs to be carefully considered if a factory-calibrated sensor is used as a nonadjunctive device. A possible trade-off between the need to improve the ease-of-use and cost of CGM technology and the need to guarantee safety and reliability would be granted by factory-calibrated systems that could eventually allow the user to perform optional calibrations, from time to time, if necessary for safety reasons.

## 5. Conclusions 

Most of the commercially available CGM sensors need to be calibrated to convert the raw measurements to glucose values. In order to preserve sensor accuracy, manufacturer instructions recommend a calibration at least every 12 h. Simple linear regression techniques have been extensively employed for calibration since the commercialization of the first CGM devices. Although their simplicity and ease-of-implementation in wearable devices represent the fundamental strength of these approaches, sensor inaccuracy problems and the need of frequent recalibrations called for the development of more sophisticated techniques, often coming from the academic community.

In the last decade, several signal processing, modelling, and machine-learning methods have been proposed from the academic community to address the calibration issue, which led to improvements in CGM sensor accuracy and user acceptability. The moderate computational complexity often facilitated the integration of such techniques in the current CGM systems, which are more accurate than the first CGM sensor generation and less calibration-dependent. The proved efficacy of some recently-proposed techniques in improving sensor performance predicts that, in the following years, more sophisticated algorithms will be integrated in next-generation CGM systems.

The increasing availability and lifetime of current- and next-generation CGM products will generate, in the upcoming years, a great amount of data, eventually available for offline processing. It is, thus, easy to expect that some recently-proposed techniques, e.g., for retrospective calibration [79,80,81,82] and for optimizing calibration parameters based on past data [77,78], would be extremely useful for post-processing CGM outputs in order to facilitate their analysis and improve performance over time by exploiting the cyclic nature of CGM use.

## Figures and Tables

**Table 1:** Summary of the reviewed calibration techniques.

Study	Calibration Technique	Model of BG-IG Dynamic	Real-Time Use in Wearable Devices	Calibrations per Day	Validation Data	Improvements Compared to Manufacturer (if Applicable)
Aussedat et al. [59]	Linear regression with feature to detect phases of steady state signal	No, but use of heuristic technique	Yes	Not specified	Real data from a miniaturized glucose sensor used in rats	/
Knobbe et al. [60,61]	Extended Kalman filter	Yes	Yes	Not specified	Real data from the Medronic (Northridge, CA, USA) MiniMed CGM system	/
Kuure-Kinsey et al. [62]	Dual rate Kalman filter	No	Yes	3	Synthetic data; data from an experimental glucose sensor used in rats	/
Facchinetti et al. [63]	Extended Kalman filter	Yes	Yes	4	Synthetic data	/
Leal et al. [64]	Auto-regressive models	No	Yes	At least 3	Real data from the Medtronic (Northridge, CA, USA) MiniMed CGMS system gold	Median RAD ^1^ decreased of 4.6%
Leal et al. [65]	Linear regression	No	No	At least 3	Real data from the Medtronic (Northridge, CA, USA) MiniMed CGMS system gold	Median RAD ^1^ decreased of 2%
Barceló-Rico [66,67]	Multiple local dynamic models [66] with adaptive parameters normalization [67]	Yes	Yes	3–4	Real data from the GlucoDay (Menarini, Florence, Italy) sensor [66]; synthetic data; real data from the Medtronic (Northridge, CA, USA) MiniMed CGMS system gold [67]	MARD ^2^ decreased of 3.9% in [66] and of 2.4% in [67]
Mahmoudi et al. [68]	Rate-limiting filtering, selective smoothing, and robust regression	No, but use of heuristic technique	Yes	Maximum 4	Real data from SCGM 1 (Roche Diagnostic, Mannheim, Germany) system	/
Kirchsteiger et al. [69,70]	Linear matrix inequalities	Yes	Yes	Roughly 6 (more in day 1)	Real data from the FreeStyle Navigator (Abbott Diabetes Care, Alameda, CA, USA) system	MARD ^2^ decreased of about 4.7% [70]
Guerra et al. [72]	Linear regression and regularized deconvolution	Yes	Yes	2	Synthetic data; real data from the FreeStyle Navigator (Abbott Diabetes Care, Alameda, CA, USA) and DexCom Seven Plus (Dexcom Inc., San Diego, CA, USA) systems	RMSE ^3^ decreased of 7.2 mg/dL
Vettoretti et al. [74]	Linear regression and regularized deconvolution	Yes	Yes	2	Real data from the Dexcom G4 Platinum (Dexcom Inc., San Diego, CA, USA) system	MARD ^2^ decreased of 1.2%
Acciaroli et al. [75]	Linear regression and regularized deconvolution	Yes	Yes	1	Real data from the Dexcom G4 Platinum (Dexcom Inc., San Diego, CA, USA) system	MARD ^2^ decreased of 1.2%, calibrations reduced from 2 to 1 per day
Acciaroli et al. [76,83]	Multiple-day model and regularized deconvolution	Yes	Yes	0.25 in [76]; zero in [83]	Real data from the Dexcom G4 Platinum (Dexcom Inc., San Diego, CA, USA) system [76] and a next-generation Dexcom prototype [83]	MARD ^2^ decreased of 1.2%, calibrations reduced from 2 to 0.25 per day [76]
Lee et al. [77]	Linear regression with run-to-run	No	Yes, after a few weeks of CGM use	2	Synthetic data	/
Zavitsanou et al. [78]	Linear regression with weakly updating feature	No	Yes, after a few weeks of CGM use	2	Real data from the Dexcom G4 Platinum (Dexcom Inc., San Diego, CA, USA) system	/
Del Favero et al. [80,81,82]	Linear regression and regularized constrained deconvolution	Yes	No	13 in [80]; 10 in [82]	Real data from the DexCom Seven Plus [80,81] and Dexcom G5 Mobile [82] (Dexcom Inc., San Diego, CA, USA) systems	MARD ^2^ decreased of 6.9% in [80], of 2.6% and 4.1% in adults and pediatrics in [82]

^1^ RAD, relative absolute difference; ^2^ MARD, mean absolute relative difference; ^3^ RMSE, root mean square error.
